# Biofilm and Antibiotic Resistance Study of Bacteria Involved in Nosocomial Infections

**DOI:** 10.7759/cureus.78673

**Published:** 2025-02-07

**Authors:** Nihal Ezzariga, Oumaima Zouhari, Amal Rhars, Zohra Lemkhente, Mohamed Aghrouch

**Affiliations:** 1 Bacteriology, Faculty of Medicine and Pharmacy, Ibn Zohr University, Agadir, MAR; 2 Nursing, Higher Institute of Nursing Professions and Health Techniques, Agadir, MAR; 3 Parasitology and Mycology, Faculty of Medicine and Pharmacy, Ibn Zohr University, Agadir, MAR; 4 Medical Biology, Centre Hospitalier Régional Hassan II, Agadir, MAR

**Keywords:** acinetobacter baumannii, aminoglycosides, antibiotic resistance, bacterial conjugation, biofilm, carbapenems, fluoroquinolones, nosocomial infection

## Abstract

Nosocomial infections are increasingly problematic due to growing bacterial resistance. Biofilms play a key role in the persistence of these infections, leading to treatment failures and poor patient outcomes. Addressing antibiotic resistance within biofilms is especially critical in hospitals, making it essential to develop new strategies to manage biofilm-related infections and curb bacterial resistance.

The study, conducted at the regional hospital center in Agadir, Morocco, analyzed 75 bacteria (37 antibiotic-sensitive and 38 resistant). Seven bacteria were isolated from catheters, and others from preserved samples. Biofilm formation was assessed using the tissue culture plate (TCP) method, involving strain recovery; culture on cystine, lactose, electrolyte-deficient (CLED) medium; microplate inoculation; staining with crystal violet; and optical density (OD) measurement.

The results showed that 77.33% of the bacteria formed biofilms. All catheter-isolated bacteria showed biofilm formation. Strong biofilm production was observed in 66.67% of *Acinetobacter baumannii* and in most *Pseudomonas aeruginosa* strains. *Enterobacteriaceae* also demonstrated significant biofilm formation. Notably, 70% of carbapenem-resistant bacteria showed strong biofilm production.

Most nosocomial bacteria form biofilms, with a higher prevalence in antibiotic-resistant strains. Sensitive bacteria also form biofilms but less frequently. Bacterial conjugation may facilitate the acquisition of carbapenem resistance within biofilms.

## Introduction

The biofilm is a complex aggregate of bacteria enclosed in a self-generated matrix of extracellular polymeric substances that provide the bacteria with a significant variety of living conditions necessary for their development and survival. It promotes interactions and the exchange of genes, including those that confer resistance to antibiotics. Biofilms form on medical devices and mucous membranes, mainly in immunocompromised patients, and are resistant to antibiotics, posing major challenges for public health. They are responsible for 60% of nosocomial infections and all infections related to prostheses [1]. Research on biofilm prevention has intensified over the past decade. The objective of this study is to evaluate biofilm formation in 75 bacterial strains responsible for nosocomial infections by exploring the relationship between antibiotic resistance and biofilm formation in order to develop new prevention and treatment strategies.

## Materials and methods

The bacterial strains studied come from various clinical samples such as protected distal specimens, cytobacteriological urine examinations, cytobacteriological pus examinations, blood cultures, and six isolated strains of catheters. In addition, a sample from a distal end of a urinary catheter infected with* Escherichia coli* was also added to the study. Bacterial strains were recovered from tubes stored at a temperature of 70°C or less, using specific codes associated with each strain. After recovery, the bacteria were regenerated by adding a small amount of the bacterial suspension to a tube containing brain heart infusion (BHI) broth, followed by incubation for 24 hours. Then, the resulting suspension was inoculated on solid media, including cystine, lactose, electrolyte-deficient (CLED) medium (and Chapman medium for *Staphylococcus*), following a precise striating process with a sterilized platinum loop. After incubation at 37°C for 24 hours, the microorganisms formed colonies visible on the solid medium. Colonies isolated after incubation were adjusted to a density of 0.5 McFarland (approximately 108 CFU/mL) in sterile physiological water. A 1/100 dilution of this suspension was prepared in sterile Mueller Hinton (MH) broth, after which the suspension was vortex-homogenized to a concentration of approximately 106 CFU/mL, ready for inoculation on microplates.

Biofilm formation was studied on 96-well microplates, where each bacterial strain was cultured in three different wells to observe and quantify biofilm formation by the tissue culture plate (TCP) method. The bacterial suspension, at a concentration of approximately 106 CFU/mL, was inoculated into the microplate wells. Wells containing sterile HD broth served as negative controls, while three ATCC strains (*E. coli* ATCC 25922, *E. coli* ATCC 35218, and *Staphylococcus aureus* ATCC 35556) were used as positive controls. All tests were performed in triplicate. After a 24-hour incubation at 37°C, the planktonic phase was gently removed from the wells, and the microplates were washed three times with physiological water to remove planktonic bacteria. The plates were then dried in an oven at 60°C for about 20 minutes, and the cells adhering to the wells were stained with 0.1% purple crystal for 40 minutes. After staining, the microplates were washed three times with distilled water and dried again. The purple crystal absorbed by the biofilms was recovered by adding 200 μL of ethanol to the wells for 20 minutes, and this solution was used to measure absorbance at 550 nm using a spectrophotometer.

Biofilm formation was classified according to optical density (OD) based on a calculated cut-off value. Biofilms were classified as non-formative, weak, moderate, or strong, according to the criteria established by Folliero et al. [2]:

Cut-off value DO = DO of the negative control (3 × standard deviation of the ODs of the negative control)

The criteria used were:

DOm ≤ threshold value OD: Non-biofilm-forming

OD DOm threshold value ≤ 2 × OD threshold value: Low biofilm formation

2 × DOm threshold value ≤ 4 × OD threshold value: Moderate biofilm formation

DOm 4 × OD threshold value: Strong biofilm formation

The data analysis was performed by applying the necessary functions in the software to interpret the OD values, and tables and graphs were plotted to visualize and interpret the results of the study.

## Results

Biofilm formation

**Figure 1:**
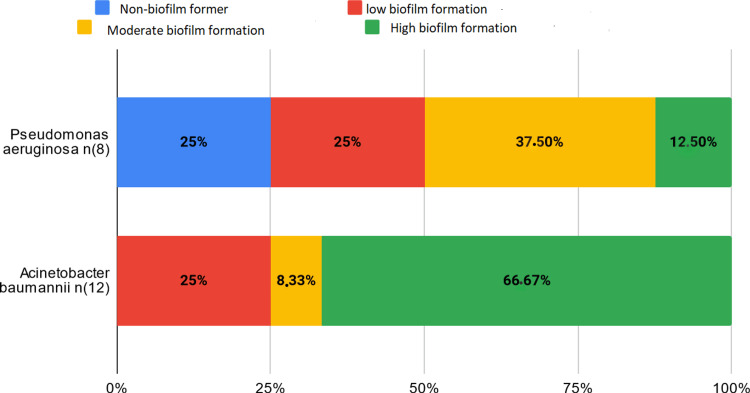
Biofilm formation in Acinetobacter baumannii and Pseudomonas aeruginosa

**Figure 2:**
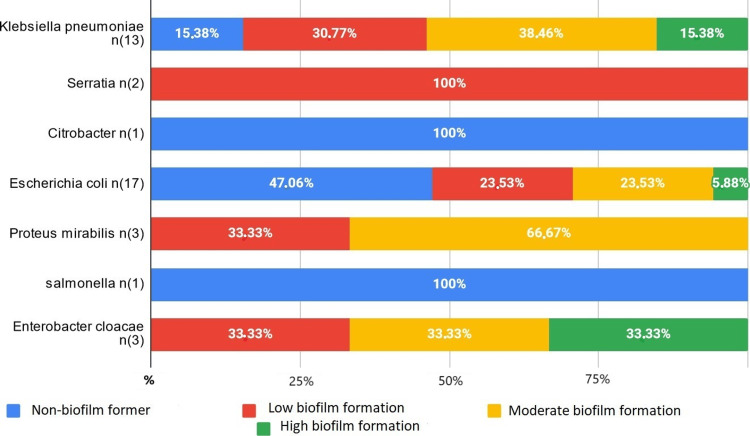
Biofilm formation in Enterobacteriaceae

**Figure 3:**
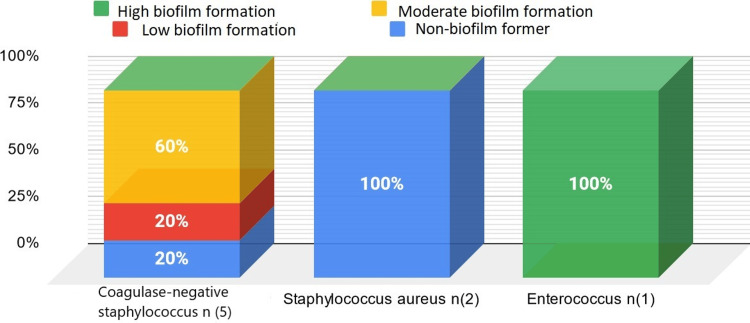
Biofilm formation in the Gram-positive cocci studied

In all, 77.33% of the bacteria studied formed a biofilm. All catheter-isolated bacteria and all *Acinetobacter baumannii* formed a biofilm, with 66.67% of them being strong formers (Figure 1). The majority of *Pseudomonas aeruginosa* formed a biofilm, with 12.5% showing strong formation (Figure 1). Among the *Enterobacteriaceae*, *Enterobacter cloacae*, *Klebsiella pneumoniae*, and *E. coli *have, respectively, 33.33%, 15.38%, and 5.88% strong formers (Figure 2). An *Enterococcus* bacterium resistant to all antibiotics showed strong biofilm formation. The two *S. aureus* studied did not form a biofilm. Among coagulase-negative *Staphylococcus*, 60% formed a moderate biofilm, 20% a weak biofilm, and 20% did not form a biofilm (Figure 3).

The correlation between antibiotic resistance and biofilm formation

**Figure 4:**
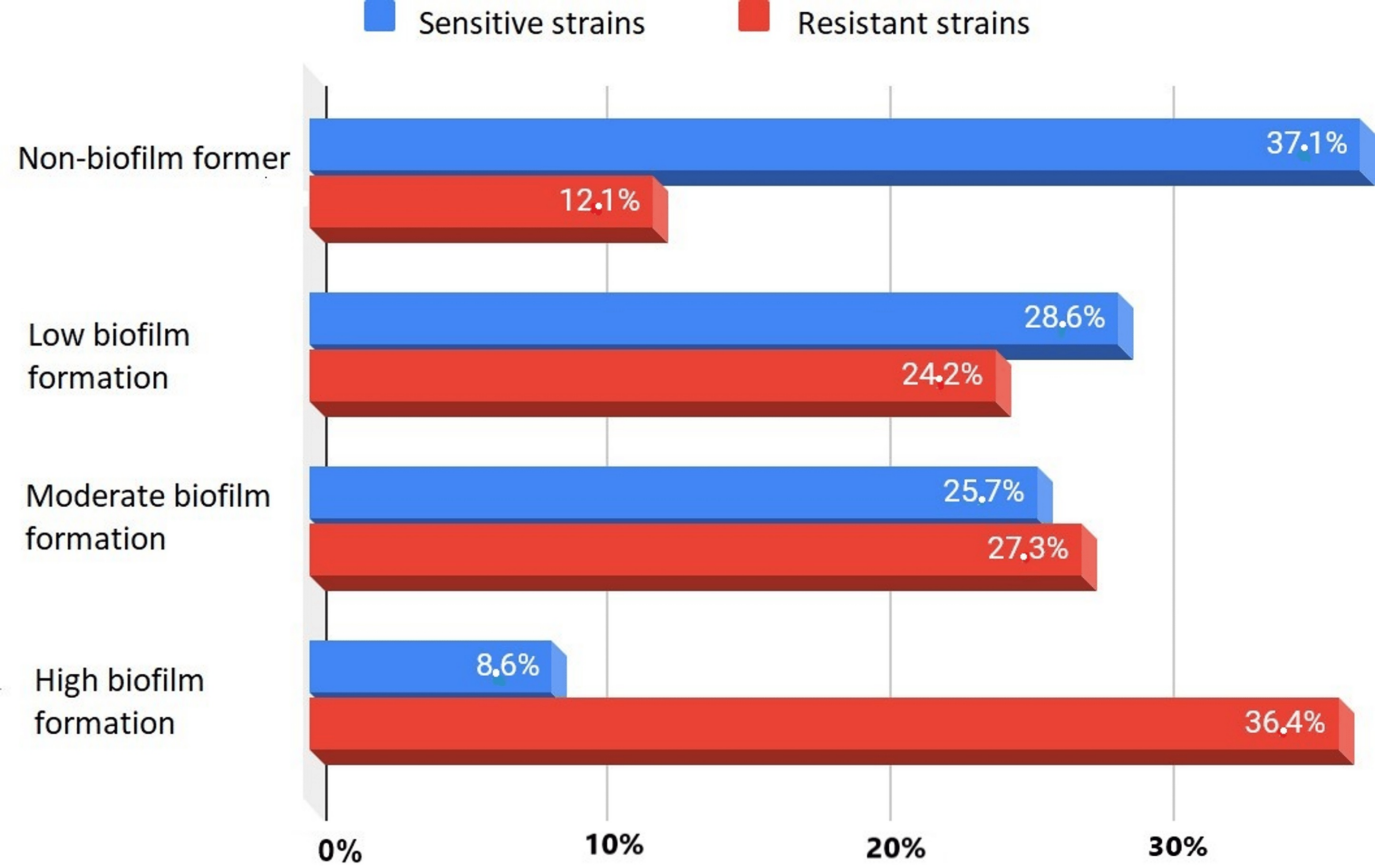
Biofilm formation in susceptible and resistant bacteria

Comparison between the results of the antibiotic susceptibility test and microplate biofilm formation showed a correlation between biofilm formation and the high rate of antibiotic resistance in bacteria since biofilm is a community in which bacteria exchange resistance genes with each other. Figure 4 illustrates the percentages of susceptible and resistant bacteria and their ability to form biofilm.

Formation of biofilm by antibiotic family

ESBL-Producing Strains

**Figure 5:**
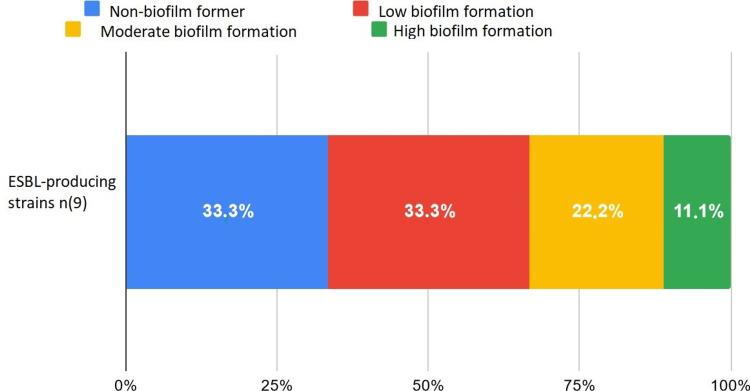
Biofilm formation in ESBL-producing bacteria

Among the ESBL-producing strains (six *K. pneumoniae* and three *E. coli*), 33.33% did not form a biofilm, 33.33% weakly formed the biofilm, 22.2% moderately formed the biofilm, and 11.1% strongly formed the biofilm (Figure 5).

Carbapenems

**Figure 6:**
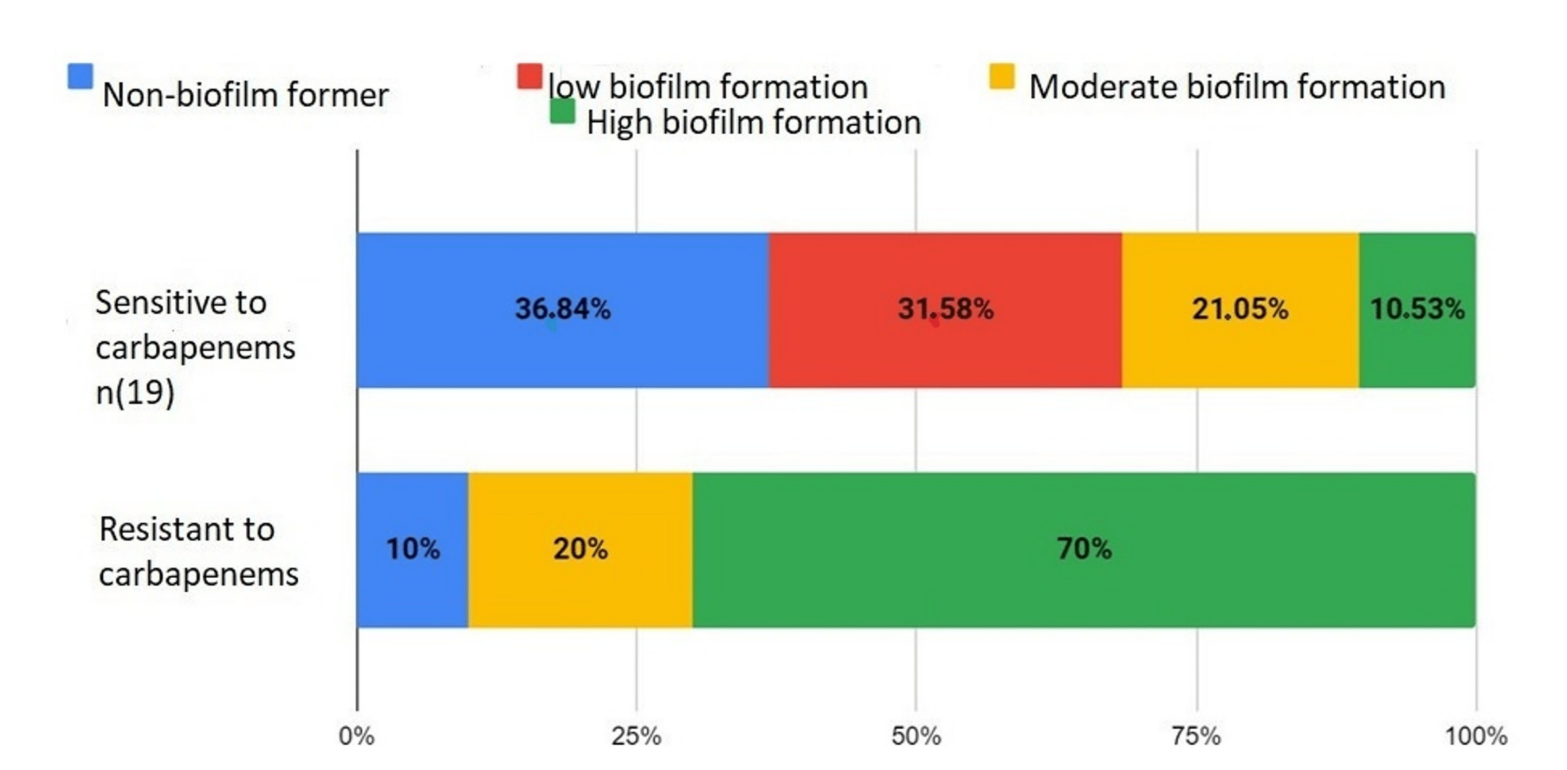
Biofilm formation in carbapenem-sensitive and resistant bacteria

In all, 36.84% of susceptible strains did not form a biofilm, 31.58% weakly formed the biofilm, 21.05% moderately formed the biofilm, and 10.53% strongly formed the biofilm. Among the resistant, 10% did not form a biofilm, 20% formed a moderate biofilm, and 70% formed a strong biofilm (Figure 6).

Fluoroquinolones

**Figure 7:**
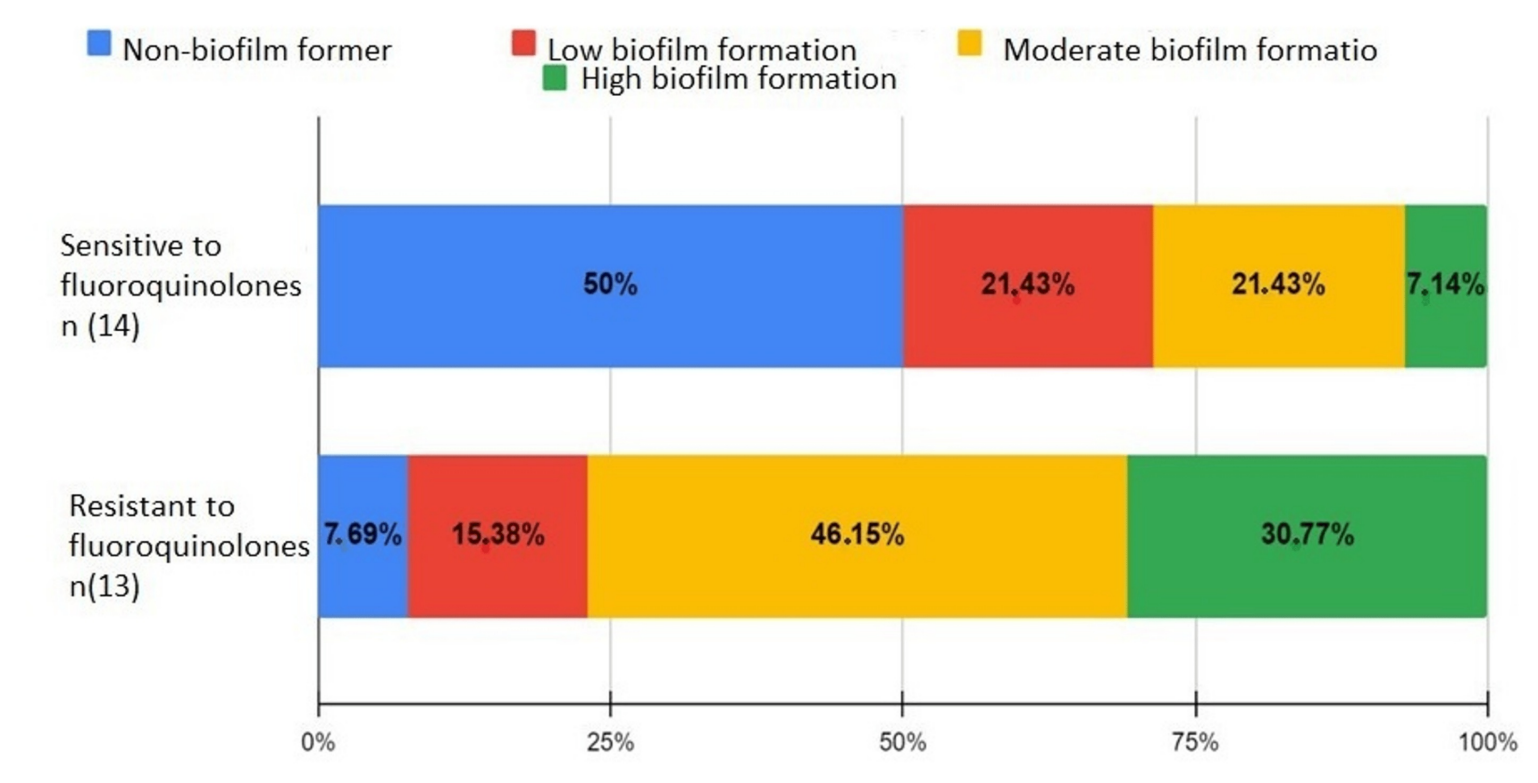
Biofilm formation in fluoroquinolone-sensitive and resistant bacteria

Of the susceptible strains, 50% did not form a biofilm, 21.43% weakly formed the biofilm, 21.43% moderately formed the biofilm, and 7.14% strongly formed the biofilm. Among the resistant, 92.31% formed a biofilm, with 15.38% showing low formation, 46.15% showing moderate formation, and 30.77% showing high formation (Figure 7).

Aminosides

**Figure 8:**
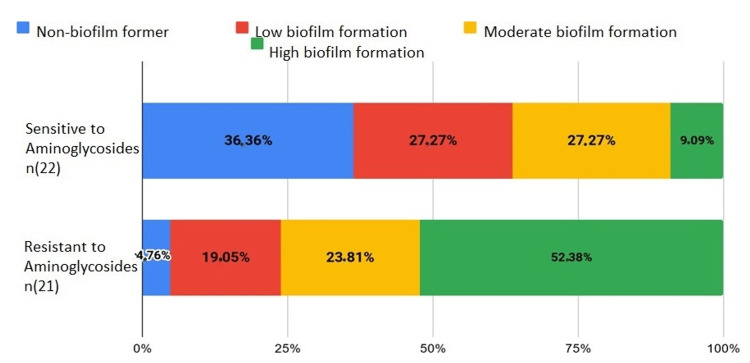
Biofilm formation in aminoglycoside-sensitive and resistant bacteria

Of the susceptible bacteria, 36.36% did not form a biofilm (*E. coli*, *S. aureus*, *Citrobacter*, and *Salmonella*), 27.27% weakly formed the biofilm, 27.27% moderately formed the biofilm, and 9.09% strongly formed the biofilm. For resistant bacteria, 4.76% did not form a biofilm, 19.05% weakly formed the biofilm (*A. baumannii* and *K. pneumoniae*), 23.81% moderately formed the biofilm (*A. baumannii*, *K. pneumoniae*, and *Enterococcus*) (Figure 8).

Multiresistance

**Figure 9:**
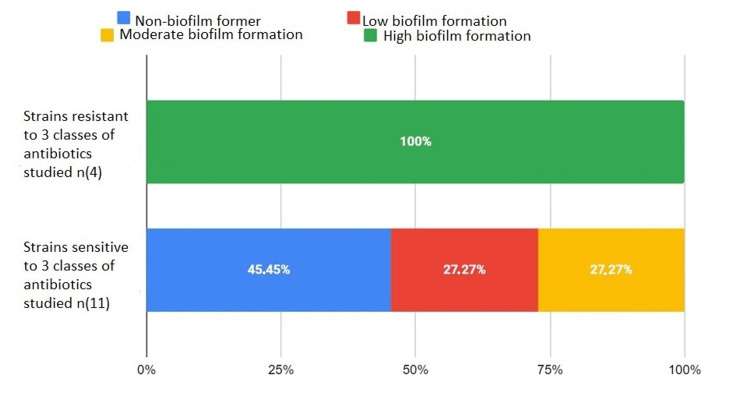
Biofilm formation in bacteria resistant to all antibiotics studied and strains susceptible to the same antibiotics

In all, 100% of the strains resistant to all antibiotics of the three families studied formed a strong biofilm (*A. baumannii* and *Enterococcus* spp). For strains sensitive to all antibiotics of the three families, 45.45% did not form a biofilm, 27.27% weakly formed the biofilm, and 27.27% moderately formed the biofilm (Figure 9).

## Discussion

Prolonged and intensive use of medical devices is associated with a high risk of infectious complications, prolonged hospitalization, and increased healthcare costs. Many patients with medical devices suffer from severe and persistent infections, often due to treatment failure [2]. According to the results of our study, all strains isolated from the seven catheters studied formed biofilms to varying degrees depending on the species. For example, one strain of *Serratia* spp. isolated from a central catheter moderately formed a biofilm, while the other two strains isolated from other biological specimens formed weak biofilms and are all sensitive to the tested antibiotics.

All strains of *A. baumannii* studied show resistance to one or more classes of antibiotics (carbapenems, fluoroquinolones, and aminoglycosides) and have formed biofilms. Multidrug-resistant strains particularly tend to form important biofilms. A study conducted in India confirmed that multidrug-resistant strains of *A. baumannii* produce more biofilms compared to susceptible strains [3].

*A. baumannii *infections constitute a significant proportion of nosocomial infections, and multidrug-resistant strains are attracting increasing attention as hospital pathogens. Recent research has shown that biofilm formation by *A. baumannii* is linked to major virulence factors, promoting bacterial persistence and drug resistance [4].

In our study, 75% of *P. aeruginosa* strains formed biofilms. Antimicrobial treatment of *P. aeruginosa* infections is often challenging due to their ability to form dense and persistent biofilms [5].

In addition, 84.62% of *K. pneumoniae* strains formed biofilms. Fimbriae type 1 or 3, capsule, and lipopolysaccharides (LPS) are virulence factors involved in this capacity [6]. Among ESBL-producing *K. pneumoniae*, 83.33% formed biofilms, indicating a relationship between biofilm formation and resistance [7].

Of the *E. coli *strains studied, 52.94% formed biofilms, mostly isolated from urine samples. The production of biofilm by *E. coli* promotes colonization of the urinary tract and complicates the treatment of urinary tract infections due to multidrug resistance [8]. An *E. coli* strain isolated from a urinary catheter showed resistance to fluoroquinolones, aminoglycosides, and ertapenem and moderately formed the biofilm, underscoring the importance of catheter removal to manage persistent urinary tract infections [9].

Regarding *S. aureus*, two isolated strains of catheters have moderately formed biofilms and are resistant to fluoroquinolones. The other two strains, which do not produce biofilm, show varied sensitivity profiles. The biofilm-forming capacity of *S. aureus* contributes to antibiotic resistance, but whether antibiotic-resistant strains have the capacity to form biofilms has not yet been determined [10].

For coagulase-negative staphylococci, 80% formed biofilms. These bacteria, commensal in humans and animals, are now recognized as infectious agents, so biofilm formation seems to be an important factor in their pathogenicity [11].

The single strain of *Enterococcus* studied, which is multidrug-resistant, formed an important biofilm. A Polish study showed that all strains of *Enterococcus* spp. isolated formed biofilms, attributing this phenomenon to a surface protein promoting colonization and biofilm formation [12].

All four strains of *E. cloacae* formed biofilms to varying degrees. A study has shown a strong association between biofilm formation and the expression of Curli-type fimbriae genes, which are important for biofilm formation and surface colonization [13].

All three strains of *Proteus mirabilis* studied formed biofilms. *P. mirabilis*, encoding 17 fimbrial operons, uses fimbriae to form functional biofilms, particularly on catheters [14].

The relationship between antibiotic resistance and biofilm formation is of great scientific interest, as these factors significantly influence infections. In all, 87.9% of bacteria resistant to one or more classes of antibiotics formed biofilms, while 37.1% of susceptible bacteria did not form a biofilm. Biofilms increase the frequency of bacterial conjugation phenomena and the chances of resistance gene transfer, making infections difficult to treat and potentially fatal [15].

The increasing incidence and prevalence of carbapenem-resistant bacteria is a global threat to human health. Biofilm formation facilitates the dissemination of resistance genes by bacterial conjugation [16].

The emergence of carbapenem resistance in *K. pneumoniae* is of concern. A significant correlation between carbapenem resistance and biofilm formation is observed, although susceptible isolates may also form biofilms [17].

For *P. aeruginosa*, biofilm formation is essential to its pathogenesis. A strain resistant to all carbapenems formed a biofilm, while susceptible strains formed weak or moderate ones. Of 82 carbapenem-resistant strains, 76 formed biofilms [18].

Regarding *E. coli*, five strains sensitive to aminoglycosides did not form a biofilm, while four resistant strains formed moderate ones. Resistance to aminoglycosides is due to enzymatic inactivation by genes on plasmids or transposons. The formation of biofilms confers tolerance to aminoglycosides [19]. One study found a correlation between gentamicin resistance and biofilm formation in *E. coli* [20].

*A. baumannii*, resistant to aminoglycosides and carbapenems, often forms biofilms, suggesting synergistic effects between these resistances. For fluoroquinolone-resistant *E. coli*, three strains formed moderate biofilms, while susceptible strains formed weak or nonexistent biofilms. Resistance is often due to mutations in the gyrA gene [21].

## Conclusions

Hospital-acquired infections, aggravated by bacterial resistance, are a major risk in the medical environment. Understanding biofilms is crucial to explaining their persistence. This study shows that most of the bacteria responsible for hospital-acquired infections can form biofilms. Although antimicrobial resistance is more common in biofilm-forming bacteria, susceptible bacteria can also form antimicrobial resistance, but to a lesser degree. Bacterial conjugation appears to be a key mechanism in the acquisition of antibiotic resistance genes within biofilms. The correlation between biofilm formation and antimicrobial resistance highlights the need to seek new therapeutic targets, including the development of anti-biofilm drugs to combat nosocomial infections.
